# Comparison of Four SARS-CoV-2 Neutralization Assays

**DOI:** 10.3390/vaccines9010013

**Published:** 2020-12-28

**Authors:** Lydia Riepler, Annika Rössler, Albert Falch, André Volland, Wegene Borena, Dorothee von Laer, Janine Kimpel

**Affiliations:** Department of Hygiene, Microbiology and Public Health, Institute of Virology, Medical University of Innsbruck, 6020 Innsbruck, Austria; lydia.riepler@i-med.ac.at (L.R.); Annika.Roessler@i-med.ac.at (A.R.); Albert.Falch@i-med.ac.at (A.F.); andre.volland@i-med.ac.at (A.V.); Wegene.Borena@i-med.ac.at (W.B.); dorothee.von-laer@i-med.ac.at (D.v.L.)

**Keywords:** SARS-CoV-2, neutralizing antibodies, neutralization assay, pseudotype virus

## Abstract

Neutralizing antibodies are a major correlate of protection for many viruses including the novel coronavirus SARS-CoV-2. Thus, vaccine candidates should potently induce neutralizing antibodies to render effective protection from infection. A variety of in vitro assays for the detection of SARS-CoV-2 neutralizing antibodies has been described. However, validation of the different assays against each other is important to allow comparison of different studies. Here, we compared four different SARS-CoV-2 neutralization assays using the same set of patient samples. Two assays used replication competent SARS-CoV-2, a focus forming assay and a TCID_50_-based assay, while the other two assays used replication defective lentiviral or vesicular stomatitis virus (VSV)-based particles pseudotyped with SARS-CoV-2 spike. All assays were robust and produced highly reproducible neutralization titers. Titers of neutralizing antibodies correlated well between the different assays and with the titers of SARS-CoV-2 S-protein binding antibodies detected in an ELISA. Our study showed that commonly used SARS-CoV-2 neutralization assays are robust and that results obtained with different assays are comparable.

## 1. Introduction

Since the first cases of SARS-CoV-2 infection have been reported in Wuhan in December 2019 the virus has spread throughout the world and caused a severe pandemic. Up to now more than 76 million infections with SARS-CoV-2 and more than 1.6 million deaths have been reported [1]. In order to control the pandemic, identifying and isolating infected patients is a crucial step. Usually, acutely infected persons are identified via qPCR testing of respiratory swab samples.

During a SARS-CoV-2 infection, patients develop adaptive immune responses against the virus. The knowledge of past infections, i.e., the serostatus of individuals or of a population, is important information as it will be an indicator for immunity. Reliable testing for the serostatus will also be important when judging the efficacy of potential vaccine candidates, the need for revaccination, or selection of plasma donors for therapy with plasma from convalescent patients. There are a great number of immunoassays such as ELISAs available for detection of SARS-CoV-2-binding antibodies [2]. Often, antibodies against the spike protein S or the nucleoprotein N are detected. Many of these assays have a high specificity and sensitivity, especially when a combination of different assays is used [3,4]. Immunoassays such as ELISAs have the advantage that they are easy to standardize and validate. Additionally, high throughput analysis of samples is possible. However, such immunoassays do not provide information on the functionality of the antibodies detected. Especially neutralizing antibodies are considered to be an important factor for immunity and potentially for the clearance of the virus in the infected individuals. The titers and kinetics of antibody production during a SARS-CoV-2 infection vary considerably and might depend on the severity of disease [5,6]. Additionally, it is not yet completely clear how long SARS-CoV-2 neutralizing antibodies last and consequently, if they can mediate long-term protection [7]. Recently, the first reinfections of convalescent patients have been reported [8,9]. Although, some studies have shown a correlation of binding and neutralizing antibodies [4,10,11], neutralizing antibody assays are still a gold standard to judge the immunity of a patient.

Different types of SARS-CoV-2 neutralization assays have been described using either replication competent SARS-CoV-2 virus [12,13] or SARS-CoV-2 spike protein (S) pseudotyped lentiviral [14] or vesicular stomatitis virus (VSV)-based particles [15,16,17]. Assays with replication competent SARS-CoV-2 isolates are normally either plaque reduction/focus forming assays or TCID_50_-based assays. However, they have the disadvantage that they require biosafety level (BSL)-3 laboratories and are often labor intense. On the other hand, assays using replication-defective pseudotyped viral particles can be performed under BSL-1 or BSL-2 conditions. However, validation of these assays against assays using replication competent SARS-CoV-2 is necessary. As all neutralization assays require living cells, they are more difficult to standardize than ELISAs and, therefore, testing of robustness of these assays is a crucial step.

Many SARS-CoV-2 studies, such as epidemiological studies, vaccine efficacy trials, trials to analyze the efficacy of plasma from COVID-19 convalescent patients as a treatment option, etc., determine neutralizing antibody titers. However, it is often difficult to compare results as different assays are used. Therefore, a thorough comparison of assays is necessary. Here, we optimize four different SARS-CoV-2 neutralization assays, two assays using replication competent SARS-CoV-2, one assay with S pseudotyped lentiviral particles, and one assay with S pseudotyped replication defective VSV particles, and determine interassay variability. Further, we compare all assays using the same standard set of COVID-19 convalescent patients.

## 2. Materials and Methods

### 2.1. Cell Lines and Plasmids

An expression plasmid encoding a C-terminally truncated, codon-optimized spike glycoprotein (pCG1-SARS-2-SΔ18) and a lentiviral vector encoding TMPRSS2 were kindly provided by Dr. Markus Hoffmann and Dr. Stefan Pöhlmann [16,18]. The truncated SARS-CoV-2 spike protein or human ACE2 were subcloned in a lentiviral vector (pLenti CMVie-IRES-BlastR, addgene #119863) for generation of stable cell lines. 293T cells (ATCC) and derivates were cultured in high glucose Dulbecco’s Modified Eagle Medium (Merck, Darmstadt, Germany) supplemented with 10% fetal bovine serum (FBS, Gibco, Carlsbad, CA, USA), 2% L-Glutamin (200 mM, Gibco), 1% Penicillin-Streptomycin (10.000 U/mL, Gibco), 1% MEM Non-Essential Amino Acids Solution (100×, Gibco), and 1% Sodium Pyruvate (100 mM, Gibco). BHK-21 cells (American Type Culture Collection, Manassas, VA, USA) were maintained in Glasgow minimum essential medium (GMEM) (Gibco) supplemented with 10% FBS, 5% tryptose phosphate broth (Gibco), and 1% Penicillin-Streptomycin. Serum adapted Vero (AC-free) (ECACC 08011101) cells were cultured in high glucose Dulbecco’s Modified Eagle Medium (Merck) supplemented with 10% FBS, 2% L-Glutamin, and 1% Penicillin-Streptomycin, for infection experiments with SARS-CoV-2 medium with 2% FBS was used. For Vero cells stably expressing TMPRSS2 (kindly provided by Dr. Markus Hoffmann and Dr. Stefan Pöhlmann) [16], medium was additionally supplemented with 10 µg/mL blasticidin. Lentiviral vectors were produced as described previously and used to generate ACE2 or S stable cell lines [19]. Cells were selected with 10 µg/mL blasticidin or 500 µg/mL hygromycin, depending on the resistance cassette of the lentiviral vector. All cell lines were cultured in humidified incubators at 37 °C and 5% CO_2_.

### 2.2. Plasma Samples and Binding Antibody Testing

Plasma samples were obtained from SARS-CoV-2 convalescent or naïve donors. Plasma samples were heat inactivated at 56 °C for 30 min and subsequently centrifuged for 5 min at 8000 rpm in a tabletop centrifuge. To determine titers of SARS-CoV-2 binding antibodies a commercially available anti-SARS-CoV-2-IgA and -IgG ELISA (Euroimmun, Lübeck, Germany) using the fully automated four-plate benchtop instrument Immunomat™ (Virion/Serion, Würzburg, Germany) for detection of anti-S IgA and anti-S IgG and an Abbott SARS-CoV-2 IgG immunoassay on the ARCHITECT i2000SR system (Abbott, Chicago, IL, USA) for detection of anti-N IgG antibodies were used. Cut-off values for all assays were used according to manufactures recommendations. For anti-S IgA and anti-S IgG antibodies an optical density (OD) <0.8 was interpreted as negative, an OD between 0.8 and 1.1 as borderline, and an OD >1.1 as positive result. The anti-N IgG immunoassay was interpreted as positive for relative light units (RLU) >1.4.

### 2.3. Viruses

The human replication competent SARS-CoV-2 was isolated from a respiratory swab sample from a qPCR positive tested patient at Medical University of Innsbruck (isolate 1.2) on Vero cells expressing TMPRSS2. The virus was propagated on Vero-TMPRSS2 cells. Cells were infected with an MOI of 0.001 and supernatant was harvested 70 h after infection, clarified by 0.45 µm filtration, and frozen at −80 °C. Viral titers were determined using focus forming or TCID_50_ assay as described below. VSVΔG viruses were generated similarly as previously described [20]. Shortly, VSVΔG viruses with GFP or luciferase as marker gene were produced on BHK-21 cells stably expressing LCMV GP. Viruses were titrated via TCID_50_ assay on BHK-21-LCMV-GP cells. For generation of SARS-CoV-2 S pseudotyped particles, subconfluent 293T cells stably expressing a C-terminally truncated version of S were infected with an MOI of 3 of the VSVΔG-GP seed stock. Then, 1.5 h after infection, inoculum was removed, cells were washed once with PBS, and fresh medium supplemented with an LCMV GP-neutralizing rabbit serum (1:200, in-house produced) was added. A total of 36 h after infection, supernatant was collected, clarified via 0.45 µm filtration, and stored in single-use aliquots at −80 °C. Lentiviral particles were generated as described previously [19]. Briefly, 10 cm dishes of 293T cells were transfected with 7.5 µg lentiviral transfer vector plasmid (encoding GFP, ACE2, TMPRSS2, or spike), 12.5 µg lentiviral Gag/Pol plasmid pCMV-dR8.91, 2 µg of VSV-G, or 4 µg of SARS-CoV-2 S encoding plasmid. Prior to transfection medium was replaced by complete medium without FBS. Then, 6 h after transfection, medium was exchanged, and complete medium was added. Supernatants were collected 24–48 h after transfection, clarified via 0.45 µm filtration, and stored at −80 °C or directly used for generation of stable cell lines.

### 2.4. Western Blotting

Western blotting was performed as previously described [21]. Proteins were detected using an anti-ACE2 antibody (R&D Systems, clone 171606, 1:5000 diluted) or an anti-tubulin antibody (Sigma, clone B-5-1-2, 1:4000 diluted) and horseradish peroxidase-conjugated secondary antibodies (mouse IgG-specific antibody from goat, Dianova, Hamburg, Germany).

### 2.5. Focus Forming Assay with Replication Competent SARS-CoV-2

For titration of virus using the focus forming assay, 96-wells containing 90% confluent Vero-TMPRSS2 cells (1.8 × 10^4^ cells seeded one day prior to the assay) were infected in duplicates with 50 µL of serial half-logarithmic diluted virus for 1 h at 37 °C. Subsequently, inoculum was removed, cells were washed once, and cultured for further 9 h in 100 µL fresh medium. After fixation for 5 min with 96% ethanol, cells were stained using serum from a SARS-CoV-2 recovered patient and a horse radish peroxidase (HRPO)-conjugated anti-human secondary antibody. The signal was developed using a 3-amino-9-ethylcabazole (AEC) substrate and the number of infected cells were counted using an ImmunoSpot S6 Ultra-V reader and CTL analyzer *BioSpot^®^ 5.0* software (CTL Europe GmbH, Bonn, Germany). Instrument settings as specified in Supplementary Table S1 were used for automatic counting. For quality control, counted well size was set to 96% to avoid background signal at the edge of the wells. Fibers were removed using the fiber removal function or manually. For neutralization assays, plasma samples were 4-fold diluted in duplicate samples in medium containing 2% FCS, starting dilution 1:16. Plasma dilutions were mixed with an equal volume of SARS-CoV-2 virus resulting in 100–200 infected cells in non-neutralized wells. Plasma/virus mixes were incubated for 1 h at 37 °C and subsequently transferred to 96-wells containing 90% confluent Vero-TMPRSS2 seeded the day before. Cells were infected with virus for 1 h at 37 °C, subsequently washed once, and cultured for further 5, 7, 8, 9, 11, or 13 h in fresh medium prior fixation and staining as described above. The 50% neutralization titers were calculated as highest plasma dilution where mean infection of duplicate samples was lower than 50% of the mean (quadruplicate samples) of control wells without plasma.

### 2.6. TCID_50_-Based Assay

To determine TCID_50_ titer, virus stocks were diluted serial 10-fold and subconfluent Vero/TMPRSS2-ACE2 cells were infected in quadruplicate samples. Plates were incubated for 48 h at 37 °C, subsequently, CPE positive wells were counted, and virus titer was calculated according to the Sperman–Kaerber formula. For neutralization assays, plasma samples were 4-fold diluted in quadruplicates and mixed with an equal volume of SARS-CoV-2 virus corresponding to 100 TCID_50_ per sample. After 1 h preincubation at 37 °C, plasma/virus mixes were transferred to 96-wells containing 1 × 10^4^ adherent Vero/TMPRSS2-ACE2 seeded the day before. Cells were incubated at 37 °C for 48 h prior evaluation of CPE via microscope. Virus dilution used for infection was retitrated in each experiment. Neutralization titers of plasma samples were determined by the highest plasma dilution protecting 50% of the infected wells.

### 2.7. VSV-Based Assay

For titration, VSVΔG-S stocks were diluted half-logarithmic in medium and subsequently used to infect subconfluent 293T-ACE2 cells seeded 1 day before. Titrations were analyzed ≈16 h after infection. For GFP-containing virus, medium was removed, and plates were analyzed using an ImmunoSpot S6 Ultra-V reader and FluoroSpot software (CTL Europe GmbH, Bonn, Germany). Instrument settings as specified in Supplementary Table S2 were used for automatic counting. For quality control, counted well size was set to 96% to avoid background signal at the edge of the wells due to remaining liquid. Fibers were removed using the fiber removal function or manually. For luciferase-containing virus, medium was aspirated, in-house produced lysis buffer (0.5% IGEPAL CA-630, Sigma and 100 mM NaCl in 10 mM Tris HCl, pH = 7.4) was added, and 30 µL cell lysate was transferred to white 96-well plates. Then, 50 µL of 50 mg/mL VivoGlo luciferin (Promega GmbH, Mannheim, Germany) were added and plates were analyzed immediately using a GloMax^®^ Discover Microplate Reader (Promega GmbH, Mannheim, Germany) and Biotek Gen5^TM^ software. For neutralization assays, plasma samples were 4-fold diluted in medium and mixed with VSVΔG-S virus with desired reporter gene. After 1 h preincubation mixes were used to infect subconfluent 293T-ACE2 cells. Plates were analyzed after ≈16 h as described above depending on the reporter gene. The 50% neutralization titers were calculated as highest plasma dilution where mean infection of duplicate samples was lower than 50% of the mean (quadruplicate samples) of control wells without plasma.

### 2.8. Lentiviral Particle-Based Assay

Lentiviral particles pseudotyped with SARS-CoV-2 S and with GFP as reporter gene were produced as described above. For titration, virus was half-logarithmic diluted in medium and subsequently used to infect subconfluent 293T-ACE2 cells in 96-wells. Two days after infection, medium was removed and the number of GFP positive cells was determined using an ImmunoSpot S6 Ultra-V reader and FluoroSpot software (CTL Europe GmbH, Bonn, Germany). For neutralization assays, plasma was 4-fold serially diluted in medium and mixed with lentiviral particles resulting in ≈100–200 GFP positive cells in virus only control wells. After 1 h of preincubation, mixes were used to infect subconfluent 293T-ACE2 cells and cells were incubated for 2 days. The 50% neutralization titers were calculated as highest plasma dilution where mean infection of duplicate samples was lower than 50% of the mean (quadruplicate samples) of control wells without plasma.

### 2.9. Statistics

Correlation analysis was performed using GraphPad Prism 8.2.0 (GraphPad Software, Inc., La Jolla, CA, USA). Not all data sets had a Gaussian distribution. Therefore, a nonparametric Spearman correlation was computed with a two-tailed *p* value and a 95% confidence interval.

## 3. Results

In this study, we established four different neutralization assays for SARS-CoV-2, a focus forming assay without virus spread and a TCID_50_ assay with multiple full virus replication cycles and spread using replication competent SARS-CoV-2 and two pseudovirus assays using replication defective VSV or lentiviral particles pseudotyped with SARS-CoV-2 spike, and compared neutralization titers obtained with these assays using a set of samples from SARS-CoV-2 convalescent patients and healthy donors.

In a first step, we characterized the set of patients using different immunoassays to determine binding antibodies to SARS-CoV-2. Table 1 summarizes titers of anti-S IgG, anti-S IgA, and anti-N IgG antibodies. Our cohort comprised samples negative in all three assays (P4 and P11), samples with discrepant results in the immunoassays (P2 and P7 with anti-S IgG positive but anti-S IgA and anti-N IgG negative results), and samples that were positive in all three immunoassays. We included both weakly positive and strongly positive samples. This set of 11 patients was subsequently analyzed in all four neutralization assays.

### 3.1. Focus Forming Unit-Assay Using Replication Competent SARS-CoV-2

**Figure 1:**
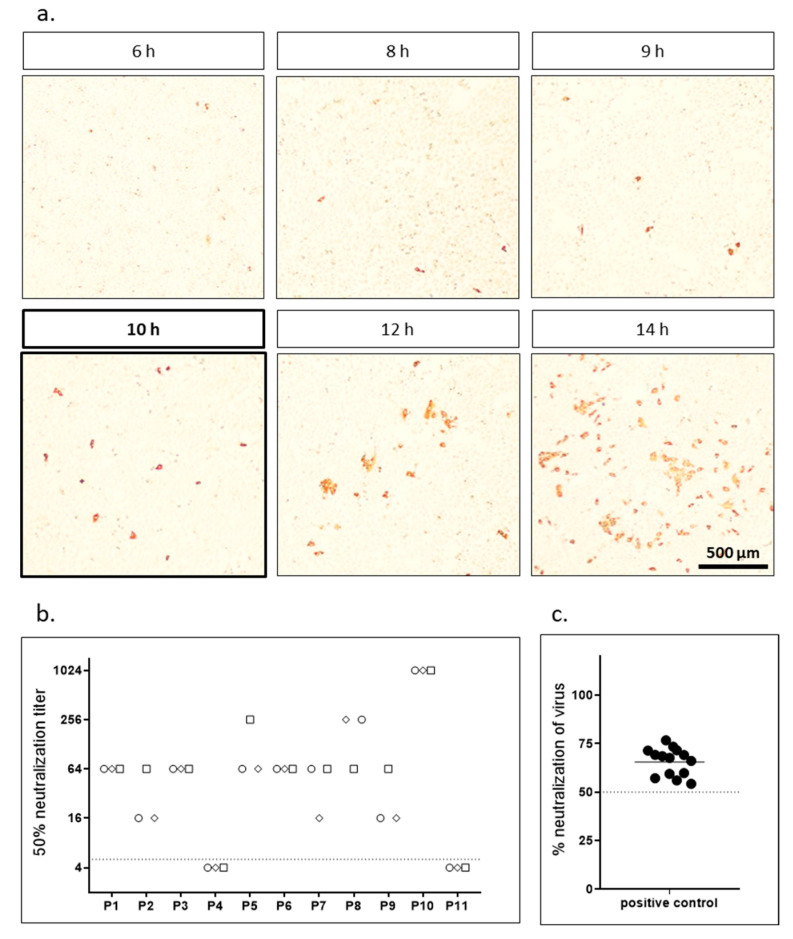
Focus forming neutralization assay. (**a**) Vero-TMPRSS2 cells were infected with replication competent SARS-CoV-2 virus, fixed after indicated incubation times and stained with the serum of a SARS-CoV-2 convalescent patient. Representative microscopic pictures are shown. (**b**) Neutralization titers for a set of 11 samples were determined using replication competent SARS-CoV-2 in a focus forming assay. Cells were fixed and stained after 10 h incubation and neutralization titers were determined using ImmunoSpot S6 Ultra-V reader and CTL analyzer BioSpot^®^ 5.0 software (CTL Europe GmbH, Bonn, Germany). The data presented are the result of three independent experiments. Each experiment is represented by a different symbol. Dotted line indicates detection limit for the assay; titers ≤ 1:4 were regarded as negative. (**c**) Low interassay variability and high reproducibility of the assay was demonstrated by analyzing neutralization titers of a positive control (P3, 1:64 dilution) in 18 independent experiment. Percent neutralization was calculated relative to virus only wells. Shown are individual replicates and mean.

For the focus forming assay we preincubated replication competent SARS-CoV-2 for 1 h with different dilutions of patient sera and subsequently infected highly susceptible Vero cells expressing TMPRSS2. After different time periods, cells were fixed and stained with the serum from a SARS-CoV-2 convalescent patient. In a first step, we determined the optimal infection time to achieve the maximal number of infected single cells before the virus started to spread and foci with several cells started to appear. As we counted infected cells automatically using an immunospot reader the goal was to obtain individual infected cells but no clusters. Figure 1a shows exemplary pictures of infected cells after different infection times. After 6 h, first infected cells were visible via immunostaining. However, the signal was weak at this time point. The intensity of the staining increased over time. After 12 h, first virus spread, and clusters of infected cells were visible. To analyze the effect of the different incubation times on neutralizing antibody titers we analyzed the set of samples shown in Table 1 in a focus forming neutralization assay (Table 2). Each sample was analyzed for five incubation times (8, 9, 10, 12, 14 h) in three independent experiments performed on different days. Variation in neutralization titers was generally low both between the different time points as well among the replicates. The 10 h time point was chosen as optimal for further experiments as it showed maximum infection without clustering and therefore allowed easy, reproducible automatic counting (Figure 1, Supplementary Figure S1). Finally, to determine interassay variability we analyzed sample P3 in a 1:64 dilution in 18 independent experiments. Figure 1c shows a high reproducibility of the assay.

### 3.2. TCID50 Assay Using Replication Competent SARS-CoV-2

**Figure 2:**
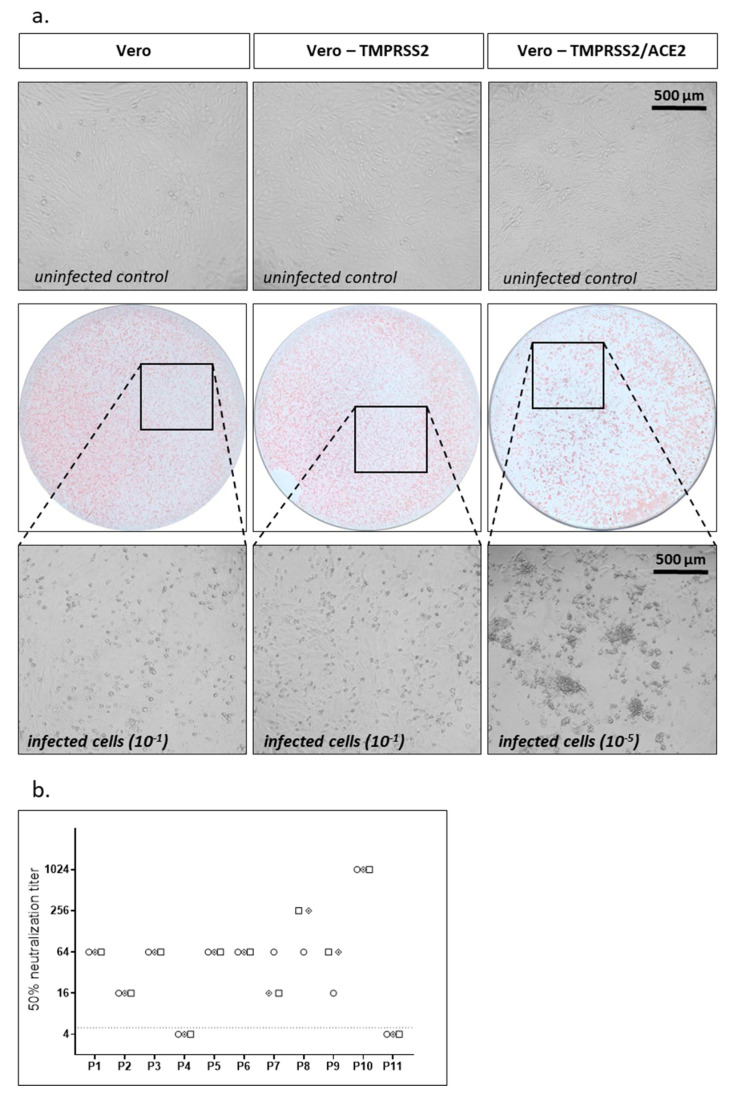
TCID_50_ neutralization assay. (**a**) Parental Vero cells, Vero cells expressing TMPRSS2, or Vero cells expressing TMPRSS2 and ACE2 were infected with serial dilutions of SARS-CoV-2. A total of 48 h after infection CPE was analyzed under the microscope and representative microscopic pictures are shown. Additionally, cells were fixed and stained with the serum of a SARS-CoV-2 convalescent patient. Upper panel: noninfected cells; middle panel: immunostaining (10^−1^ dilution for Vero and Vero-TMPRSS2 cells, 10^−5^ dilution for Vero-TMPRSS2/ACE2 cells); lower panel: higher magnification of middle panel to evaluate CPE. (**b**) Neutralization titers for a set of 11 samples were determined using replication competent SARS-CoV-2 in a TCID_50_ assay. Neutralization titers of three independent experiments are displayed with different symbols. Dotted line indicates detection limit for the assay; titers ≤ 1:4 were regarded as negative.

As a second assay using replication competent SARS-CoV-2 we performed a TCID_50_-based assay. In a first step, we analyzed cytopathic effects (CPE) of our SARS-CoV-2 isolate on Vero cells expressing TMPRSS2 or parental Vero cells, which was not very prominent (Figure 2a). To enhance susceptibility of cells to SARS-CoV-2 replication we overexpressed ACE2, the receptor for SARS-CoV-2, using a lentiviral vector. The resulting Vero-TMPRSS2/ACE2 cells stably overexpressed TMPRSS2 and ACE2 simultaneously via one lentiviral vector encoding TMPRSS2 and a blasticidin resistance cassette and a second one encoding ACE2 and a hygromycin resistance cassette. Cells were selected with blasticidin and hygromycin to maintain expression of both transgenes. While parental Vero cells only expressed marginal amounts of ACE2, stable Vero-TMPRSS2/ACE2 cells expressed high levels of ACE2 (Supplementary Figure S2a). Overexpression of ACE2 indeed made Vero-TMPRSS2 cells more susceptible to SARS-CoV-2 infection as the same virus had a ≈1 log increased titer on Vero cells overexpressing TMPRSS2 and ACE2 compared to Vero cells only expressing TMPRSS2 when titrating the virus in a focus forming assay (data not shown). In parallel, the CPE on Vero-TMPRSS2/ACE2 cells was also drastically enhanced (Figure 2a).

For the neutralization assay we incubated 100 TCID_50_ of SARS-CoV-2 with different dilutions of patient sera for 1 h, infected Vero-TMPRSS2/ACE2 cells, and after 2 days analyzed CPEs. We determined the 50% neutralization (TCID_50_) titers for our standard set of samples in three independent experiments using the TCID_50_-based assay (Figure 2b). As before for the focus forming assay, interassay variability was low for the TCID_50_-based assay.

### 3.3. VSV-Based Assay

Although both assays using replication competent SARS-CoV-2 produced highly reproducible results, their need of aBSL-3 laboratory limits applicability. Therefore, we established assays using replication defective VSVΔG or lentiviral particles pseudotyped with SARS-CoV-2 S, which can be performed under BSL-2 conditions.

**Figure 3:**
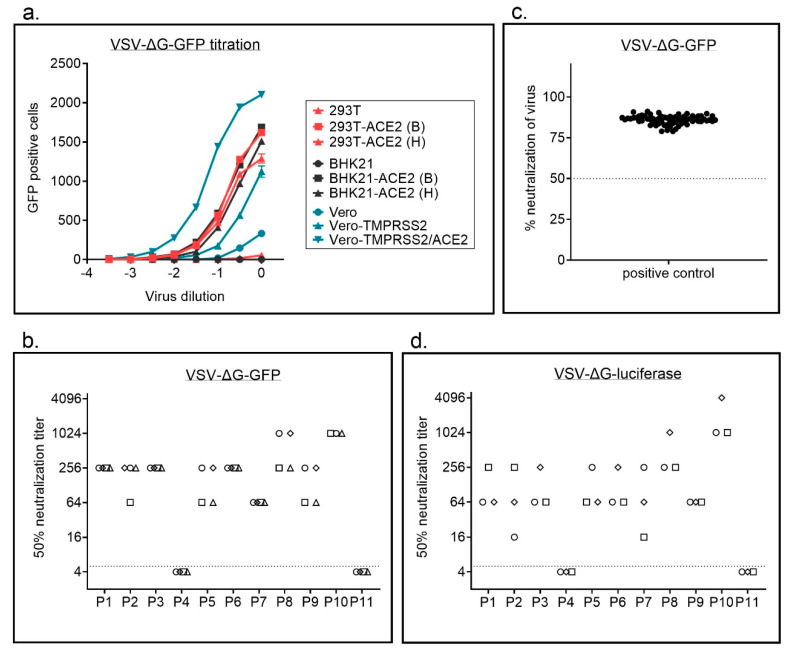
Vesicular stomatitis virus (VSV)-based neutralization assay. (**a**) VSVΔG-GFP-S particles were titrated on parental 293T, BHK21, and Vero cells or on cell lines with stable ACE2 overexpression. For 293T and BHK-21 cells two different lentiviral vectors were used for ACE2 overexpression, either with a blasticinin resistance (B) or a hygromycin resistance cassette (H). A total of 16 h after infection, the number of GFP positive cells was determined using an immunospot reader. Shown are mean ± SEM of duplicate samples from one representative of two independent experiments. (**b**) Neutralization titers for a set of 11 samples were determined using a GFP encoding VSVΔG-S variant. Each sample was analyzed in four independent experiments. Each experiment is represented by a different symbol. Neutralization titers ≤ 1:4 are regarded negative. (**c**) A positive control (P3 in a 1:64 dilution) was analyzed using the VSVΔG-GFP-S virus on 66 independent plates. Percent neutralization was calculated relative to virus only wells. Shown are individual replicates and mean. (**d**) Neutralization titers for a set of 11 samples were determined using a luciferase encoding VSVΔG-S variant. Each sample was analyzed in three independent experiments. Each experiment is represented by a different symbol. Dotted line in b + d indicate threshold for neutralizing antibody titers, i.e., titers ≤ 1:4 are regarded as negative. Dotted line in c indicates 50% neutralization.

In a first step, we compared titers for replication defective VSVΔG particles pseudotyped with SARS-CoV-2 full length or C-terminally truncated spike protein of SARS-CoV-2and found higher titers for the C-terminally truncated variant (Supplementary Figure S3). To facilitate production of replication defective VSVΔG particles pseudotyped with SARS-CoV-2 S we next established a BHK-21 cell line stably expressing the C-terminally truncated S variant (SΔ18), which has previously also by others been shown to produce better titers for lentiviral and VSV-based particles [13]. We used these cells to produce VSVΔG particles with different reporter genes, GFP or luciferase. VSVΔG-GFP-S particles, expressing GFP as a marker gene, were titrated on different cell lines (Figure 3a, Supplementary Figure S2b). We used wild type 293T with a low or absent expression of ACE2 and hamster BHK-21 cells, which do not express human ACE2 and are consequently not susceptible to SARS-CoV-2 infection. The same cells were also transduced with lentiviral vectors to overexpress human ACE2, the receptor for SARS-CoV-2, either in combination with a blasticidin (B) or a hygromycin (H) resistance cassette. For the African green monkey cell line Vero we used three different variants; (i) parental cells, (ii) cells overexpressing TMPRSS2, for which an enhanced infectability has been shown previously, and (iii) a newly generated variant simultaneously overexpressing TMPRSS2 and ACE2. While parental 293T and especially BHK-21 cells were only poorly infected by S pseudotyped VSV particles, overexpression of ACE2 strongly enhanced infection. As seen in replication competent SARS-CoV-2 virus, overexpression of ACE2 enhanced susceptibility of Vero cells expressing TMPRSS2 also for VSVΔG-S particles. Infectability of cells with SARS-CoV-2 correlated with ACE2 expression (Supplementary Figure S2).

In a next step, we compared VSVΔG variants encoding different reporter genes, GFP or luciferase, for the neutralization assay. All assays were performed on 293T-ACE2 cells. Automatic counting of GFP positive cells using an immunospot reader worked well (Supplementary Figure S4). GFP and luciferase encoding viruses resulted in similar neutralization titers with a low interassay variability (Figure 3b,d, Supplementary Figure S5). For the GFP encoding virus we analyzed patient P3 in a 1:64 dilution in 66 independent experiments and also saw a high reproducibility of the assay (Figure 3c).

### 3.4. Lentiviral Vector Based Assay

**Figure 4:**
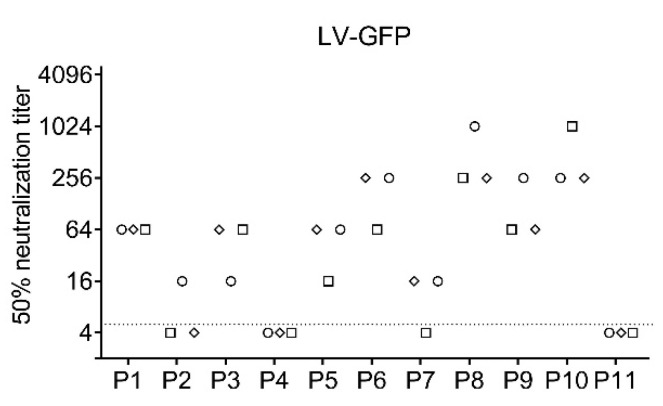
Lentiviral particle-based neutralization assay. Lentiviral particles encoding GFP were pseudotyped with SARS-CoV-2 S. Neutralization titers for a set of 11 samples were determined. Each sample was analyzed in three independent experiments. Each experiment is represented by a different symbol. Dotted line indicates detection limit for the assay; titers ≤ 1:4 were regarded as negative.

As a second BSL-2 compatible system we used lentiviral particles pseudotyped with SARS-CoV-2 S expressing GFP as reporter. Again, a C-terminally truncated S variant was used to increase virus titers. Patient sera were preincubated with the virus and subsequently 293T cells expressing ACE2 were infected. As for the VSVΔG, GFP positive cells could be counted with the immunospot reader. However, automatic counting was more difficult as infected cells divided (Supplementary Figure S6). The same set of patients as before was analyzed for the lentiviral particle-based SARS-CoV-2 neutralization assay in three independent experiments (Figure 4). The titers obtained with the lentiviral particles were more variable compared to the other systems, especially for weakly positive samples such as P2 and P7.

### 3.5. Comparison of Assays

**Figure 5:**
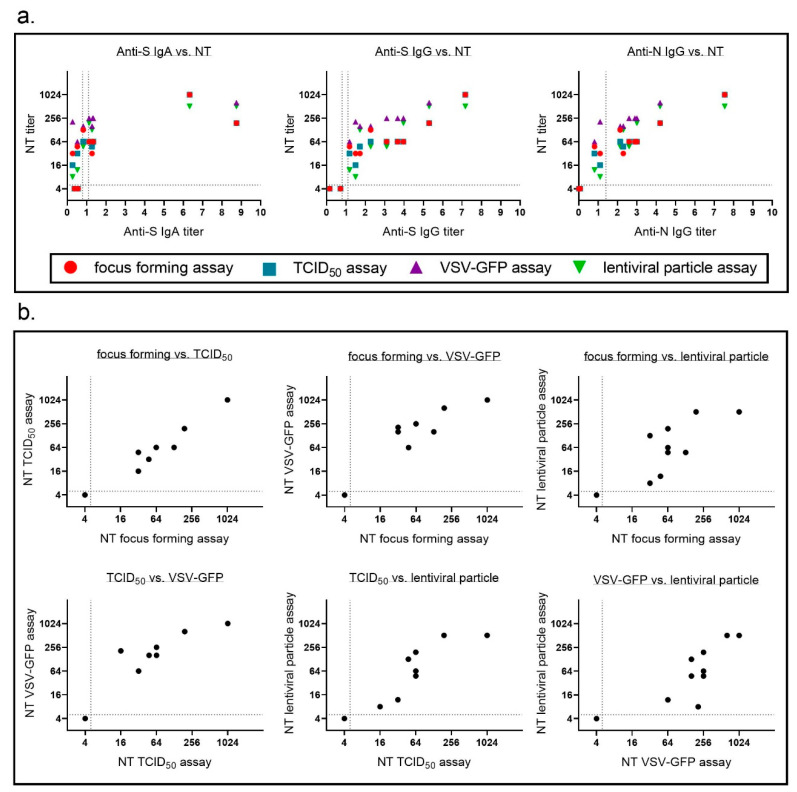
Neutralization titers for the different assays correlate well with each other and with binding antibody titers. For neutralization assays mean neutralizing titers of three (focus forming, TCID_50_, and lentiviral particle assay) or four independent experiments (VSV assay) were calculated. (**a**) Correlation of neutralizing antibody titers and binding antibodies (anti-S IgA, anti-S IgG, and anti-N IgG) were determined. (**b**) Neutralizing antibody titers determined in each assay were correlated to titers in all three other neutralizing antibody assays. Dotted lines indicate detection limit for the different assays; neutralization assay ≤ 1:4 negative, anti-S IgA and anti-S IgG OD < 0.8 negative, OD 0.8–1.1 borderline, OD > 1.1 positive, anti-N IgG RLU > 1.4 positive. Some of the dots in the blots represent multiple samples.

We showed that each of the four SARS-CoV-2 neutralization assays we used was highly reproducible and had a low interassay variability. Neutralizing antibody titer obtained with each of the assays correlated well with each of the immunoassays for binding antibodies (Figure 5a, Table 3). The most interesting question in this study was how comparable the different neutralizing antibody assays were. As different labs use different assays to determine SARS-CoV-2 neutralizing antibody titers it is crucial to compare neutralization titers obtained with all assays using a standard set of samples. Therefore, in a last step, we compared neutralization titers for our standard patient samples for all assays (Figure 5b). We correlated each assay with the other three assays and determined Spearman’s r (Table 4). Neutralization titers for all four assays correlated well with each other with a tendency for lower correlations with the lentiviral particle-based assay. Regarding sensitivity all assays seemed to perform equally well with a consistent detection even of samples with low levels of binding antibodies.

## 4. Discussion

In the present study we established and compared four different SARS-CoV-2 neutralization assays, a focus forming and a TCID_50_-based assay using replication competent SARS-CoV-2, and two pseudovirus assays, one based on VSV and the other based on lentiviral particles. As many different SARS-CoV-2 neutralization assays are commonly used it is very important to cross reference these different assays. This will allow a better comparison of studies performed in different labs. We found that results for all four neutralization assays correlated well with each other. Similar to others, we also found a correlation of binding IgG and neutralizing antibodies.

Pseudotyped virus assays have the advantage that they do not require BSL-3 laboratories and are therefore easier to establish and allow higher throughput. When analyzing the efficacy of vaccine candidates based on chimeric VSV variants there is a potential risk of cross-reactivity of antibodies against the vaccine vector with the pseudotyped VSV used for the neutralization assay. However, as the pseudovirus particles lack an endogens envelope glycoprotein and SARS-CoV-2 spike protein is the only viral protein responsible for neutralization this cross reactivity is highly unlikely.

Similar to others we also saw an increased titer for VSV-based particles pseudotyped with a C-terminally truncated version of the spike protein compared to a full-length spike (data not shown) [13,18]. This is in accordance with previous reports for other coronaviruses showing that truncation of spike protein enhances incorporation into viral particles [22,23]. The C-terminus of coronavirus spike protein contains an ER retention signal, which is most likely the reason that its deletion enhances transport of spike protein to the cell surface and incorporation into viral particles that bud from the cell surface such as lentiviruses and VSV.

Pseudotyped viruses are also a valid tool for identification of alternate receptors of SARS-CoV-2 or cofactors enhancing infection without the need of a BSL-3 facility. Similar to others, we observed an increased susceptibility of cells for the SARS-CoV-2 spike pseudotyped VSV when human ACE2 was overexpressed. Recently, also TMPRSS2 and Neuropilin-1 have been described as cofactors enhancing SARS-CoV-2 entry [16,24].

In our study, both assays using pseudotyped viruses produced reproducible results, which correlated well with binding antibody titers obtained in the immunoassays and neutralizing antibody titers determined with replication competent SARS-CoV-2. Patient plasma samples were reproducibly positive or negative in all assays, with the exception of the lentiviral particle-based assay, where two patients gave positive neutralization titers in some experiments and negatives in others. In general, we saw a slightly lower correlation of the other test systems with the assay using lentiviral particles than for the VSV-based assay. This might be explained by the readout of the number of GFP positive cells using an immunospot reader. As lentiviral particles, in contrast to the VSV particles, integrate, the GFP signal is maintained upon cell division leading to clusters of GFP positive cells for lentiviral particles. As we also saw for the replication competent SARS-CoV-2 virus, clusters of cells are more difficult to count using automatic counting in the immunospot reader.

Remarkably, the two patients with discrepant results regarding neutralizing antibodies (P2 and P7) had also discrepant results in the immunoassays and were positive for anti-S IgG but negative for anti-N IgG indicating a weak immunity to SARS-CoV-2 in these patients. In our previous epidemiological study in an Austrian SARS-CoV-2 hotspot the majority of patients with discrepant immunoassays were positive for neutralizing antibodies [11]. In our current study also the VSV-based assay had a high sensitivity in patients with discrepant immunoassay results.

A further advantage of assays using pseudotyped viruses is that different variants of SARS-CoV-2 spike protein can easily be used for pseudotyping and therefore can be compared regarding their neutralization sensitivity. Mutations attributed to resistance to certain SARS-CoV-2 monoclonal neutralizing antibodies have recently been described [25,26]. A panel of different pseudotyped VSV variants could be used to map resistance in human plasma samples. This approach could also be helpful to analyze if different vaccine approaches, e.g., full length spike protein versus single domains, induce different types of neutralizing antibody responses. Resistance to certain neutralizing antibodies as well as generally low antibody titers could allow reinfections, as recently described [8,9]. Further studies will be needed to determine threshold titers of neutralizing antibodies needed for protection. However, all four assays described here result in robust and reproducible neutralization titers and are therefore good candidates for further studies.

## Figures and Tables

**Table 1:** Titers of binding antibodies.

Patient	Anti-S IgG ^§^	Anti-S IgA ^§^	Anti-N IgG ^$^
P1	3.669 (positive)	1.345 (positive)	2.90 (positive)
P2	1.493 (positive)	0.275 (negative)	1.10 (negative)
P3	3.103 (positive)	1.317 (positive)	2.60 (positive)
P4	0.715 (negative)	0.375 (negative)	0.00 (negative)
P5	2.275 (positive)	0.827 (borderline)	2.14 (positive)
P6	3.970 (positive)	1.130 (positive)	3.00 (positive)
P7	1.183 (positive)	0.523 (negative)	0.81 (negative)
P8	5.298 (positive)	8.745 (positive)	4.20 (positive)
P9	1.722 (positive)	1.283 (positive)	2.30 (positive)
P10	7.180 (positive)	6.325 (positive)	7.54 (positive)
P11	0.156 (negative)	0.558 (negative)	0.07 (negative)

^§^ <0.8 negative; 0.8–1.1 borderline; >1.1 positive; ^$^ >1.4 positive.

**Table 2:** Neutralization titers for focus forming assay after different infection times.

Patient	8 h			9 h			10 h			12 h			14 h		
P1	64	64	256	64	256	64	64	64	64	64	64	64	256	256	64
P2	16	16	16	16	64	16	16	64	16	16	16	16	16	64	64
P3	64	256	64	64	64	64	64	64	64	64	64	64	256	256	16
P4	4	4	4	4	4	4	4	4	4	4	4	4	4	4	4
P5	64	64	64	64	16	64	64	256	64	64	64	256	64	256	16
P6	16	64	64	64	64	64	64	64	64	256	64	64	64	256	64
P7	16	64	16	64	64	664	64	64	16	64	64	16	64	64	16
P8	64	256	256	256	64	64	256	64	256	64	256	256	256	1024	256
P9	16	64	16	16	64	64	16	64	16	64	64	16	16	64	16
P10	256	1024	1024	1024	1024	1024	1024	1024	1024	1024	1024	1024	1024	1024	1024
P11	4	4	4	4	4	4	4	4	4	4	4	4	4	4	4

**Table 3:** Comparison binding antibody titer and neutralization titer.

Comparison		Spearman’s r	95% Confidence Interval	*p* Value ^§^
Anti-S IgA	Focus forming	0.7558	0.2664–0.9354	0.0093
Anti-S IgA	TCID_50_	0.8674	0.5435–0.9665	0.0010
Anti-S IgA	VSV-GFP	0.7927	0.3498–0.9460	0.0053
Anti-S IgA	Lentiviral particle	0.8513	0.4985–0.9622	0.0015
Anti-S IgG	Focus forming	0.9033	0.6507–0.9759	0.0003
Anti-S IgG	TCID_50_	0.9654	0.8632–0.9916	<0.0001
Anti-S IgG	VSV-GFP	0.9586	0.8382–0.9899	<0.0001
Anti-S IgG	Lentiviral particle	0.9291	0.7344–0.9825	<0.0001
Anti-N IgG	Focus forming	0.8526	0.5022–0.9625	0.0014
Anti-N IgG	TCID_50_	0.9420	0.7788–0.9858	<0.0001
Anti-N IgG	VSV-GFP	0.9586	0.8382–0.9899	<0.0001
Anti-N IgG	Lentiviral particle	0.9520	0.8141–0.9883	<0.0001

^§^ two-tailed.

**Table 4:** Comparison of neutralization assays.

Comparison		Spearman’s r	95% Confidence Interval	*p* Value ^§^
Focus forming	TCID_50_	0.9693	0.8782–0.9926	<0.0001
Focus forming	VSV-GFP	0.8388	0.4650–0.9588	0.0019
Focus forming	Lentiviral particle	0.8121	0.3967–0.9514	0.0035
TCID_50_	VSV-GFP	0.9055	0.6576–0.9765	0.0002
TCID_50_	Lentiviral particle	0.9063	0.6600–0.9767	0.0003
VSV-GFP	Lentiviral particle	0.8515	0.4992–0.9622	0.0015

^§^ two-tailed.

## Data Availability

The data presented in this study are available in the article and supplementary material.

## Supplementary Materials

The following are available online at https://www.mdpi.com/2076-393X/9/1/13/s1, Figure S1: Exemplary wells for focus forming assay after counting using an immunospot reader, Figure S2: ACE2 expression determines infectability of cells with VSVΔG-S particles, Figure S3: A C-terminally spike variant produces higher titers of pseudotyped VSV vectors compared to full length spike, Figure S4. Exemplary wells for VSV-based neutralization assay after counting using an immunospot reader, Figure S5. Inhibition curves for VSV-based neutralization assay, Figure S6. Exemplary wells for lentiviral particle-based neutralization assay after counting using an immunospot reader, Figure S7. Inhibition curves for lentiviral particle-based neutralization assay.
