# Supplementary material for: Comparison of Four SARS-CoV-2 Neutralization Assays
Source: Vaccines (Basel). 2020 Dec 28;9(1):13. doi: 10.3390/vaccines9010013 (PMC7824240; doi:10.3390/vaccines9010013)
Supplement: Supplementary file 1 [file vaccines-09-00013-s001.pdf]

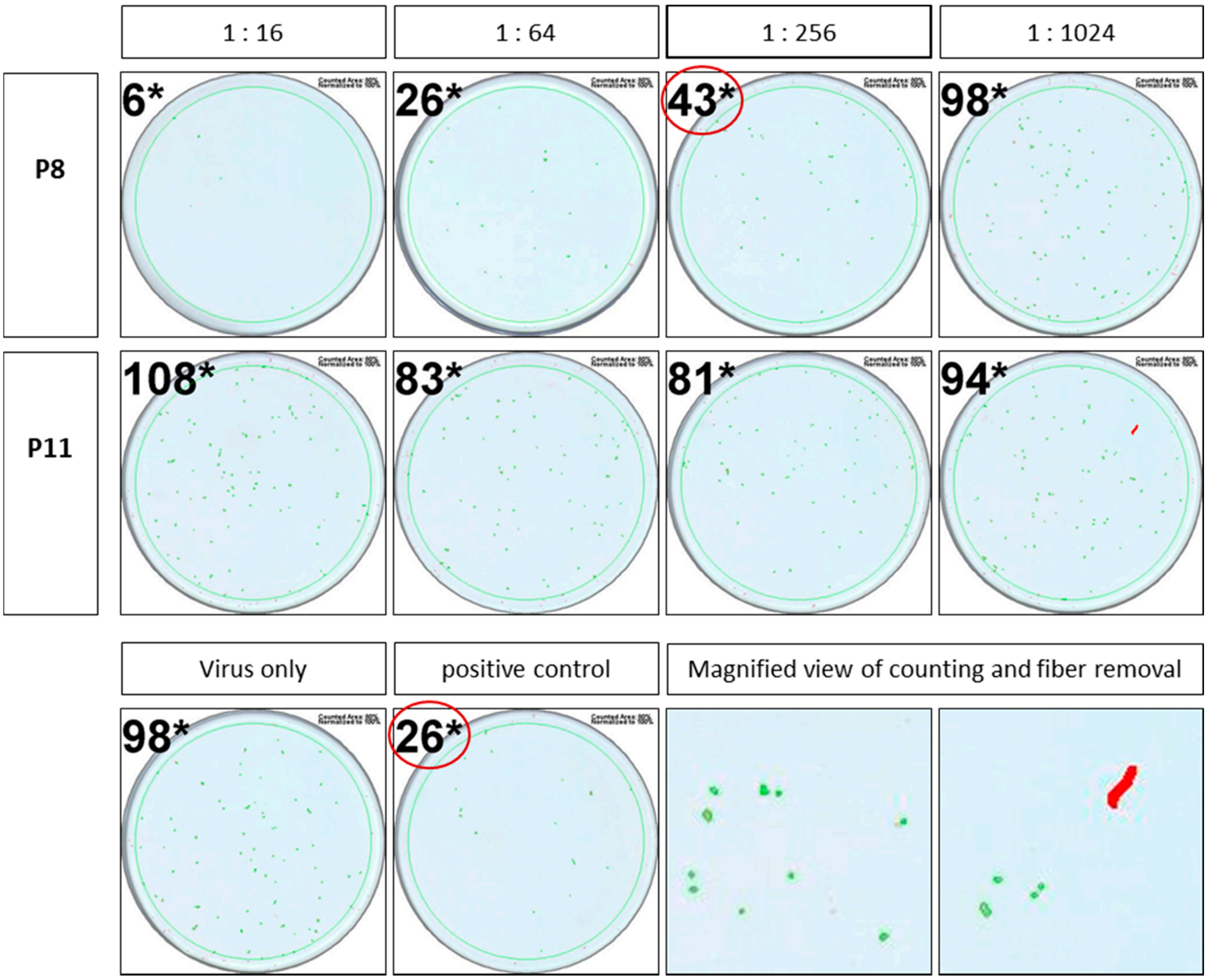

Supplementary Figure 1. Exemplary wells for focus forming assay after counting using an immunospot reader. Shown are representative wells for the focus forming assay for a neutralizing (P8) and a non-neutralizing (P11) sample in different plasma dilutions, a virus only and a positive control (P3 in 1:64 dilution) well. Total counted area is marked by a green circle, counted spots are circled in green and excluded events (fibers etc.) are marked in red. For each well the number of spots after quality control (removal of fibers etc.) is given. A magnified view for counting and fiber removal (4.3-fold magnification) are shown. Counted events are marked with a green circle, removed events (fibers) are shown in red.

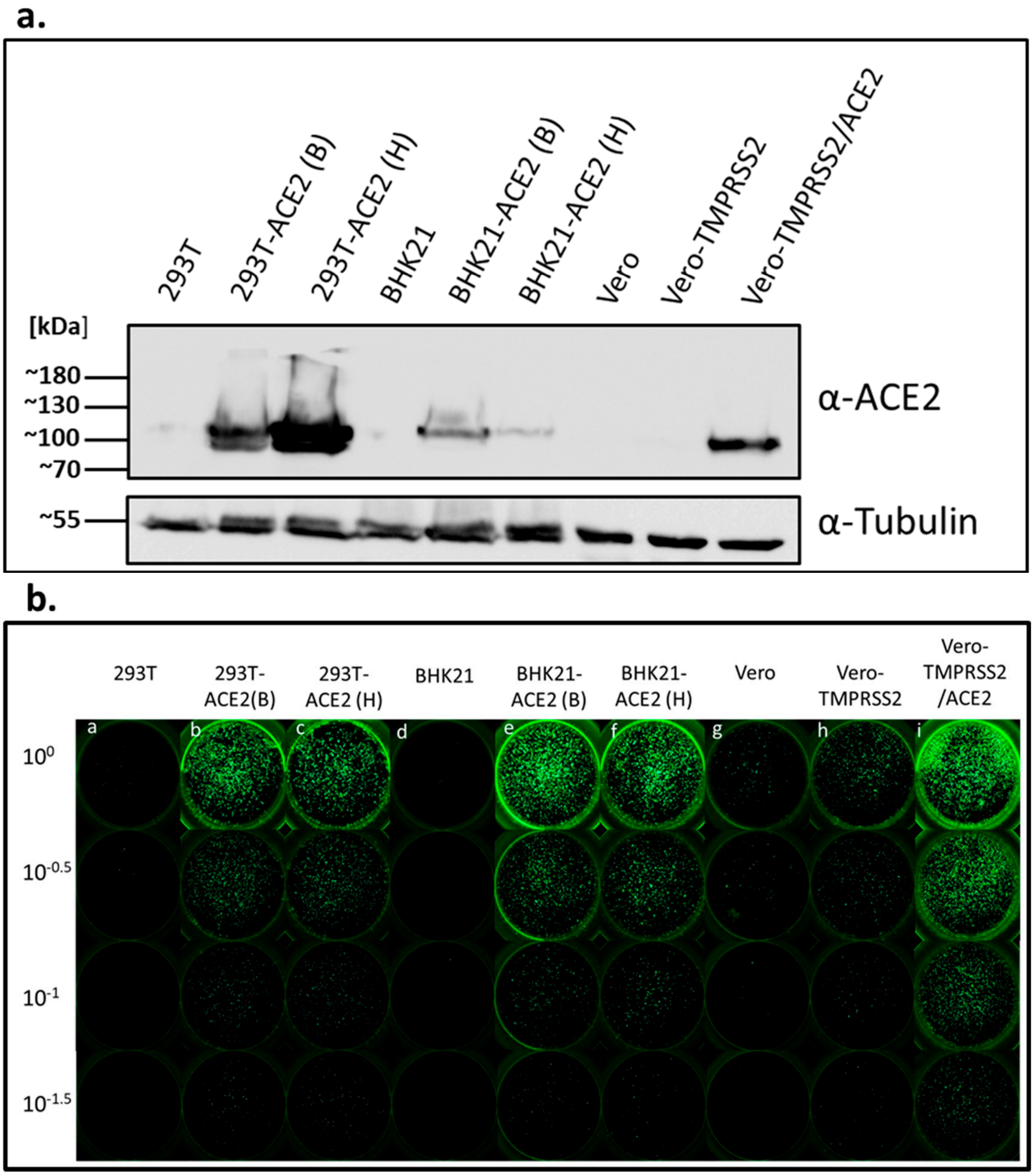

Supplementary Figure 2. ACE2 expression determines infectability of cells with VSVΔG-S particles. Cells were transduced with lentiviral vectors encoding ACE2 (B with blasticidin resistance, H with hygromycin resistance) and selected with the corresponding antibiotic. (a) Expression of ACE2 was confirmed using western blot analysis (upper blot: anti-ACE2 antibody, lower blot: anti-tubulin antibody as loading control). (b) Cells were infected with serial dilutions of VSVΔG-GFP particles pseudotyped with SARS-CoV-2 spike in 96 wells. Readout of pseudotyped VSV titer was performed 16 hours post infection using an immunospot reader.

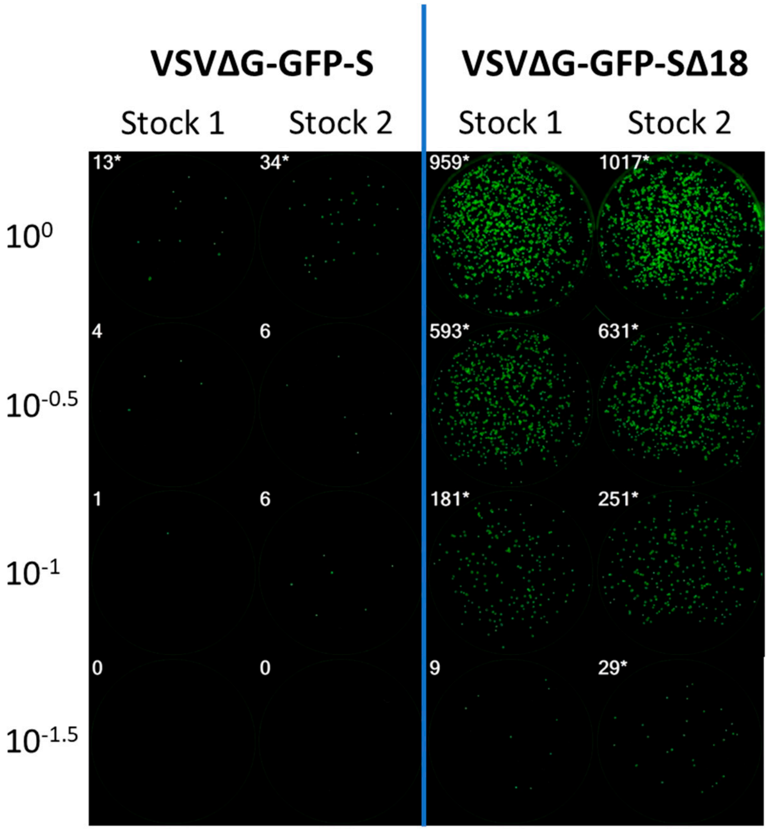

Supplementary Figure 3. A C-terminally spike variant produces higher titers of pseudotyped VSV vectors compared to full length spike. VSVΔG-GFP particles were either pseudotyped with full length (VSVΔG-GFP-S) or a C-terminally truncated version (VSVΔG-GFP-SΔ18) of SARS-CoV-2 spike protein on transiently transfected cells. For each virus variant two independent stocks were produced. Viruses were titrated on 293T-ACE2 cells in 96 wells in triplicate samples. Readout of pseudotyped VSV titer was performed 16 hours post infection using an immunospot reader. Shown are representative exemplary images for each triplicate. For each well the number of spots after quality control (removal of fibers etc.) is given.

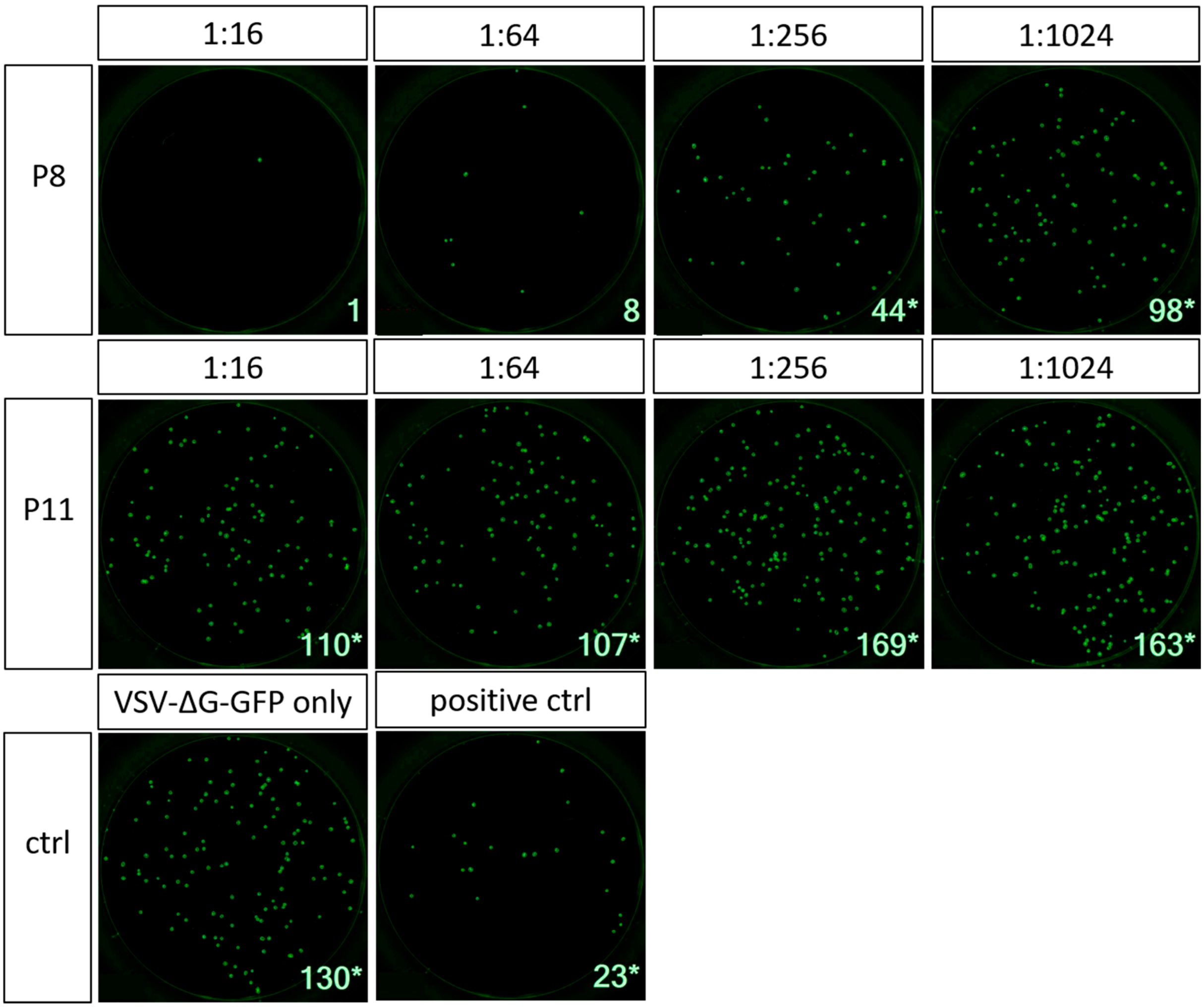

Supplementary Figure 4. Exemplary wells for VSV-based neutralization assay after counting using an immunospot reader. Shown are representative wells for the VSV-based neutralization assay for a neutralizing (P8) and a non-neutralizing (P11) sample in different plasma dilutions, a virus only and a positive control (P3 in 1:64 dilution) well ~16 h after infection. For each well the number of GFP positive spots after quality control (removal of fibers etc.) is given.

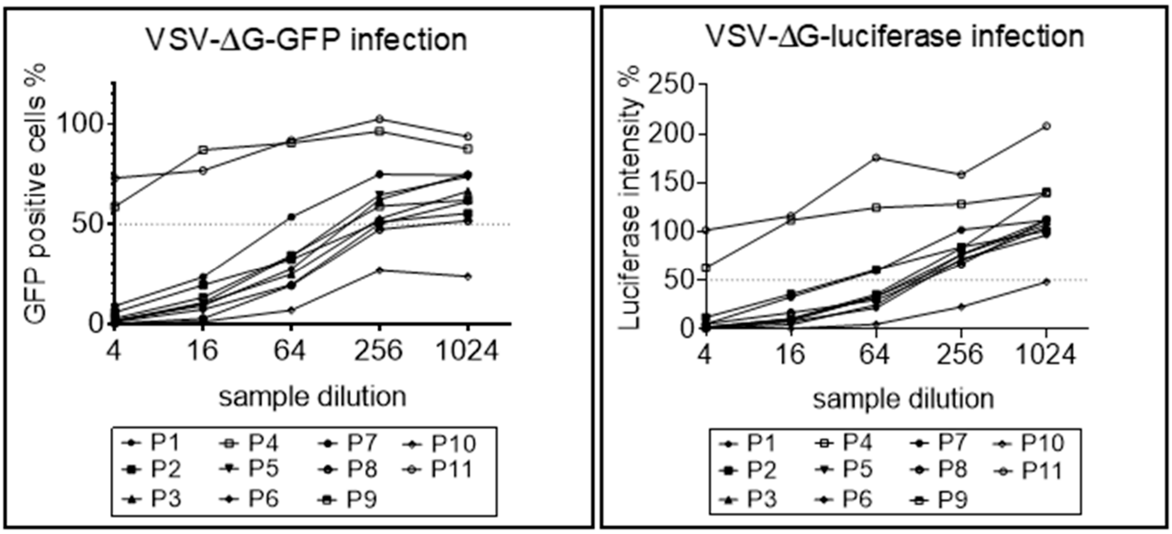

Supplementary Figure 5. Inhibition curves for VSV-based neutralization assay. For each patient plasma dilution, percent GFP positive cells or percent luciferase intensity relative to virus only wells was calculated. Exemplary data from one representative experiment is shown for GFP-encoding and luciferase-encoding VSVΔG particles. Dotted lines indicate 50 % inhibition.

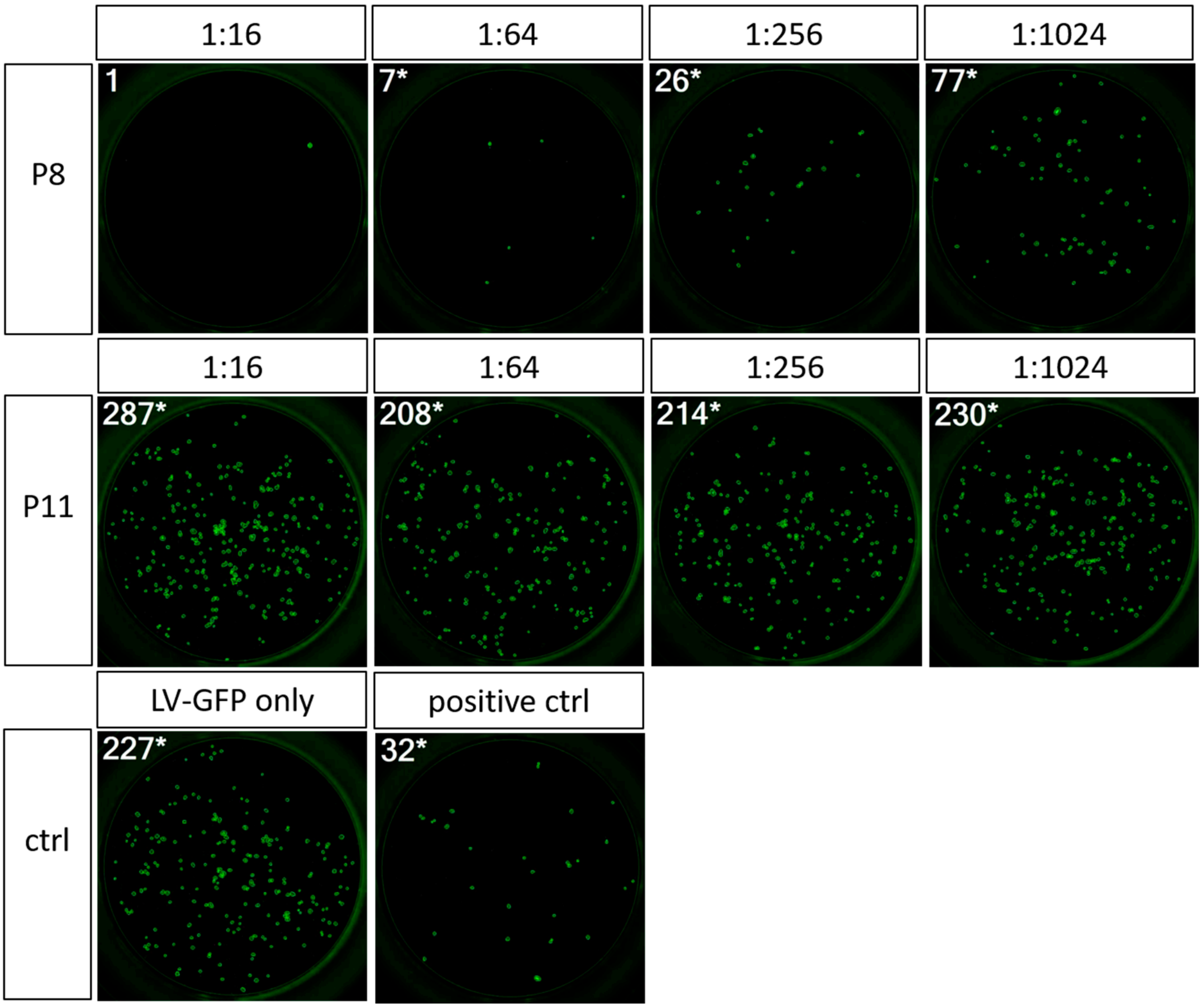

Supplementary Figure 6. Exemplary wells for lentiviral particle-based neutralization assay after counting using an immunospot reader. Shown are representative wells for the lentiviral particle-based neutralization assay for a neutralizing (P8) and a non-neutralizing (P11) sample in different plasma dilutions, a virus only and a positive control (P3 in 1:64 dilution) well. For each well the number of GFP positive spots after quality control (removal of fibers etc.) is given.

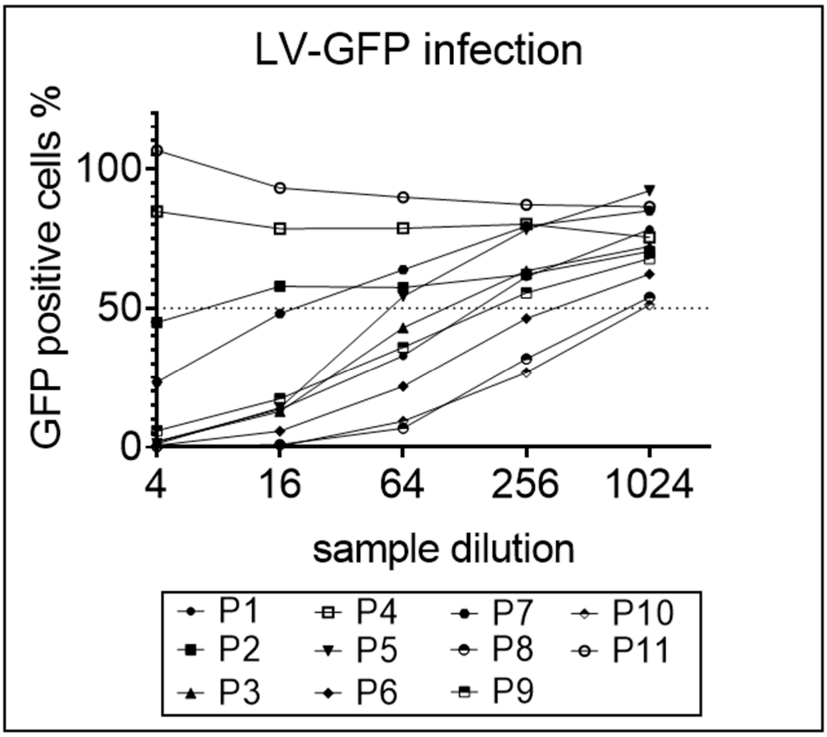

Supplementary Figure 7. Inhibition curves for lentiviral particle-based neutralization assay. For each patient plasma dilution percent GFP positive cells relative to virus only wells was calculated. Exemplary data from on representative experiment is shown for GFP-encoding lentiviral particles. Dotted line indicates 50 % inhibition.

Supplementary Table 1: Instrument settings for counting of focus forming assay

Software version: BioSpot 7.0.20.0	
Counting window size: 600x600	
Assay: 1	Edge Compensation Before PP: On
Counting Mask Size(%): 96	Ring-wise Compensation: Off
Normalize Counts of Mask: On	Edge Separation: Off
Sensitivity: 242	Sensitivity Offset: 50
Min. SpotSize: 0.0002 Sq.mm	Border Radius Percentage [%]: 30.0
Max. SpotSize: 0.0201 Sq.mm	Border Outline Percentage [%]: 80.0
Oversized Spots were Estimated	Spot Circularity Filtering: Off
Spot Separation: 9.00	Area Weight: 0
Diffuseness: Small	Circularity Threshold: 1.5
Overdeveloped Area Handling was Active	Maximum Area of Filtered Spots [pix]: 500.0
(Auto Areas: Estimated, Manual Areas: Normalized)	Use Global Object Enhancement: Off
Objects: Detailed (dark spots over light background)	Method: ES: Enhanced Separation
Background Balance was On	Aperture [pix]: 20
Background Balance: 40	Weight: 3.0
Fill Holes was Off	Enhanced Separation Level: 0
Hair Removal was On	
Audit Spots was Inactive	
ROI-Touching Removal was Off	
Edge Effect Compensation: Off	
Weight Function Shape: 0.5	
Edge Compensation Level: 1.0	

Supplementary Table 2: Instrument settings for counting of VSV and lentivirus assay

Counting Parameters	Pairing Parameters
2_520	Common/Algorithm Specific Parameters
Counting Mask Size(%): 96	Elongated spot radius ratio tolerance: 1.5
Normalize Counts of Mask: On	Maximum spots' center of mass distance (% of well bottom diameter): 0.75
Smart Count(TM): On Default Sensitivity: 145	Overlay dilation iterations: 2
Min. SpotSize: 0.0012 Sq.mm	Spot circularity threshold: 2
Max. SpotSize: 9.8222 Sq.mm	Common/Counting Window
Oversized Spots were Normalized	Height: 600
Spot Separation: 0.00	Width: 600
Diffuseness: Large	
Overdeveloped Area Handling was Active	
(Normalized)	
Objects: Inverted Normal	
Background Balance was On	
Background Balance: 40	
Fill Holes was Off	
Hair Removal was Off	
Audit Spots was Inactive	
